# Effects of Biopsychosocial Model-Based Patient Education on Pain and Pain-Related Risk Factors After Total Knee Arthroplasty: A Retrospective Propensity Score-Matched Study

**DOI:** 10.7759/cureus.78707

**Published:** 2025-02-07

**Authors:** Junji Nishimoto, Naoki Deguchi, Shigeharu Tanaka, Yu Inoue, Ryo Tanaka

**Affiliations:** 1 Graduate School of Humanities and Social Sciences, Hiroshima University, Higashi-Hiroshima, JPN; 2 Department of Rehabilitation, Saitama Medical Center, Saitama Medical University, Kawagoe, JPN; 3 Research Team for Promoting Independence and Mental Health, Tokyo Metropolitan Institute for Geriatrics and Gerontology, Itabashi, JPN; 4 Department of Rehabilitation, Faculty of Health Sciences, Tokyo Kasei University, Sayama, JPN; 5 Department of Physical Therapy, School of Health Science and Social Welfare, Kibi International University, Takahashi, JPN

**Keywords:** biopsychosocial model, knee osteoarthritis, pain, patient education, total knee arthroplasty

## Abstract

Background: The impact of interventions based on a biopsychosocial (BPS) model, including components related to sleep and nutrition, on pain after total knee arthroplasty (TKA) remains unclear. The purpose of this study was to develop patient education (PE) based on the BPS model and to clarify its effects on pain after TKA.

Methods: Participants were 121 patients who had undergone unilateral TKA for knee osteoarthritis. Patients who received usual physiotherapy (control group, n = 71) or usual physiotherapy plus PE (PE group, n = 50) were identified. The primary outcome was the change in Knee injury and Osteoarthritis Outcome Score (KOOS) pain score from baseline to three months post-TKA.

Results: After propensity score matching, there was no statistically significant difference in the change in KOOS pain scores between the control groups and PE (*p* = 0.143, *r* = 0.240). Regarding pain-related risk factors, the Central Sensitization Inventory (*p* = 0.041, *r* = 0.238), Pittsburgh Sleep Quality Index (*p* = 0.040, *r* = 0.239), and Pain Catastrophizing Scale (*p* = 0.004, *r* = 0.334) scores improved statistically significantly more in the PE group than in the control group. Hospital Anxiety and Depression Scale (HADS)-Anxiety (*p* = 0.233, *r* = 0.139), HADS-Depression (*p* = 0.333, *r* = 0.113) were not statistically significantly different between the two groups.

Conclusions: A BPS model-based PE was developed, and its effects on pain and pain-related risk factors were clarified. PE may improve central sensitization, sleep disturbance, and pain catastrophizing, which are key pain-related risk factors.

## Introduction

Despite the generally high effectiveness of total knee arthroplasty (TKA), approximately 10-34% of patients who undergo TKA experience chronic post-surgical pain (CPSP) [1, 2]. CPSP, as defined by the International Association for the Study of Pain, is pain persisting for ≥3 months after surgery [3]. The number of TKA procedures performed annually is increasing in developed countries [4, 5], and there is concern that the number of patients with residual pain after TKA will also increase. Persistent pain after TKA is a primary predictor of postoperative dissatisfaction; thus, reducing pain after TKA is essential [6].

CPSP is caused by inadequate management of acute pain; thus, interventions regarding nociceptive pain, in addition to central sensitization, are required. For chronic pain such as CPSP, approaches based on cognitive behavioral therapy (CBT) and biopsychosocial (BPS) models are being explored [7, 8]. CBT is not aimed at directly treating pain but rather seeks to alter the relationship between behaviors and reinforcing factors in chronic pain. Previous studies have demonstrated its effectiveness in reducing pain intensity and disability [9]. The approach in the BPS model enables patients to learn to control their internal environment (pain-related thoughts and feelings) and influence their reactions to the external environment (physical condition, various stresses) through education, CBT, relaxation training, and active adaptation [10]. One approach based on the BPS model is pain neuroscience education (PNE), which focuses on educating patients about the neurobiological and neurophysiological processes involved in pain [11]. A previous study demonstrated the effectiveness of PNE in treating patients with chronic pain after nonsurgical treatment [12]. However, in another study, PNE was ineffective in reducing pain in patients who underwent TKA [12]. PNE usually targets central sensitization, and the failure to consider acute nociceptive pain may have contributed to the ineffectiveness of PNE in reducing pain after TKA [11, 13].

The inclusion of information on sleep and nutrition in patient education (PE) may help reduce pain. Improvements in sleep disturbances are effective in reducing pain in the early postoperative period [14, 15]. Central sensitization and sleep disturbances are risk factors for CPSP after TKA [15, 16]. Dietary therapy reduces inflammation and nociceptive pain associated with chronic pain [17]. As such, appropriate nutritional management may also improve nociceptive pain and prevent the transition to chronic pain [17]. Therefore, interventions focusing on sleep and nutrition are important for preventing pain.

However, the impact of PE, including content on sleep and nutrition, on improving patients' pain after TKA remains unknown. The impact of interventions focusing on nociceptive pain and central sensitization on improving pain in patients with TKA is also unclear, and addressing these issues may help prevent pain after TKA.

The purpose of this study was to develop a PE based on a BPS model that includes content related to sleep and nutrition and to determine the effect of an individualized intervention during the acute postoperative period on pain and pain-related risk factors after TKA. We hypothesized that BPS model-based PE would improve pain and pain-related risk factors after TKA.

## Materials and methods

Ethics

This study was conducted in accordance with the principles of the Declaration of Helsinki and approved by the Institutional Review Board of the author’s institution (approval number: 2021-169). This study is registered with the University Hospital Medical Information Network (approval number: UMIN000046741).

Study design

This study was a propensity score-adjusted retrospective cohort study. The follow-up was conducted three months after TKA to assess the effects of PE post-TKA on pain, central sensitization, sleep disturbance, pain catastrophizing, anxiety, and depression.

Setting

This study was conducted at the Department of Rehabilitation, Saitama Medical Center, Saitama Medical University. A series of assessments and treatments were performed on patients who underwent TKA between April 2021 and March 2024. Physical therapists from the rehabilitation department recruited potential participants.

Participants

The inclusion criteria were as follows: (1) knee osteoarthritis (OA), (2) unilateral TKA, and (3) able to walk independently with or without the use of assistive devices before and after TKA. The exclusion criteria were as follows: (1) Mini-Mental State Examination score (MMSE) ≤ 23 (2) rheumatoid arthritis, (3) systemic lupus erythematosus, (4) psychiatric disorders, (5) neurological problems (paralysis or stroke), (6) post-TKA complications (deep vein thrombosis or fracture), and (7) revision TKA.

**Figure 1:**
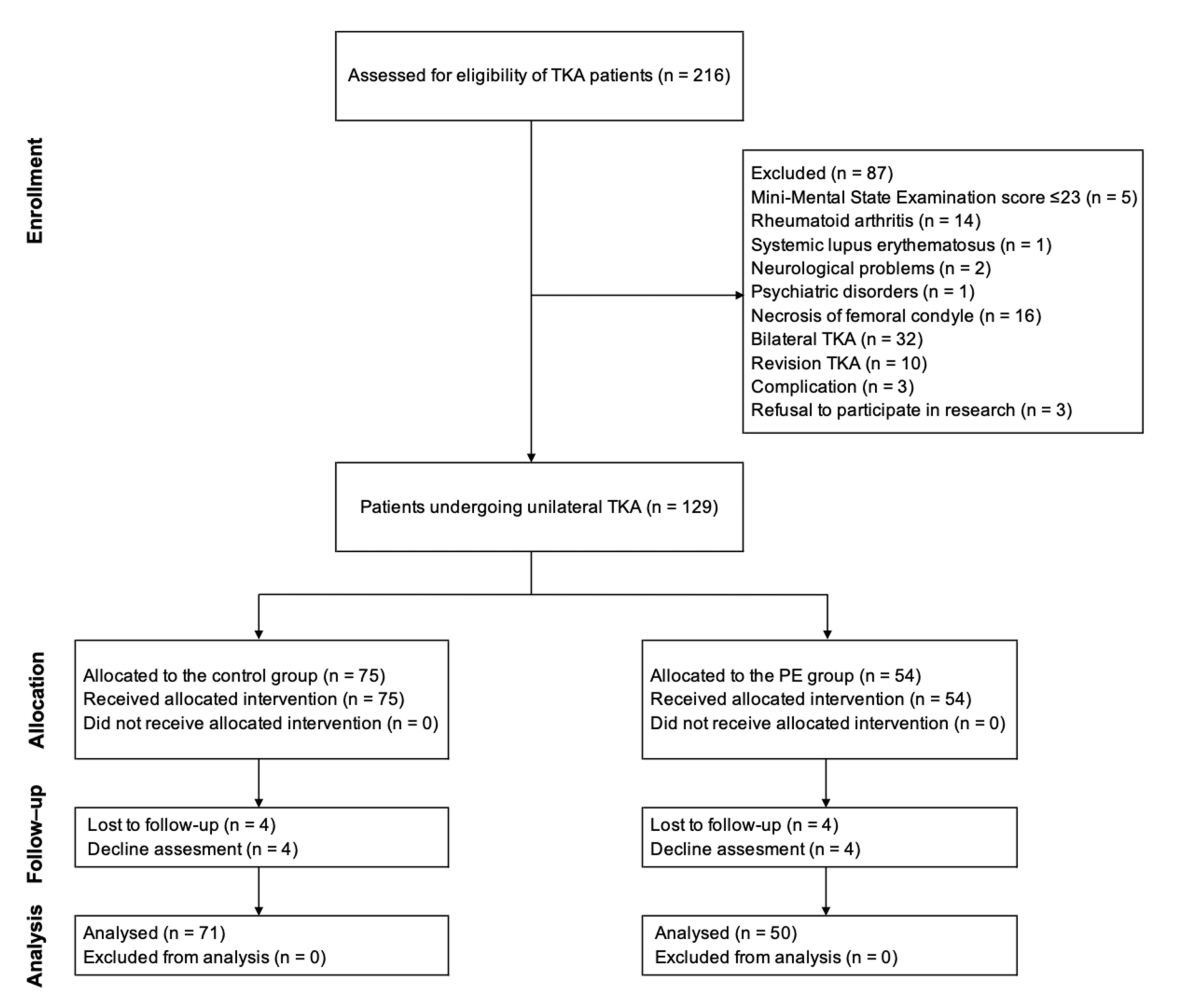
Flow diagram. PE, patient education; TKA, total knee arthroplasty.

Of the 216 patients who underwent TKA, 87 were excluded, leaving 129 eligible for follow-up. Patients were excluded from the study for the following reasons: MMSE ≤ 23 (n = 5), rheumatoid arthritis (n = 14), systemic lupus erythematosus (n = 1), neurological problems (n = 2), psychiatric disorders (n = 1), necrosis of femoral condyle (n = 16), bilateral TKA (n = 32), revision TKA (n = 10), complications (n = 3), and refusal to participate in research (n = 3). Eight patients were lost to follow-up and were excluded from the analysis. Thus, 121 patients (71 in the control group and 50 in the PE group) completed the final evaluation three months after TKA. The flowchart for patient inclusion is shown in Figure 1. From April 2021 to October 2022, patients in the control group received conventional physical therapy only, and from November 2022 to March 2024, patients in the PE group received conventional physical therapy plus PE.

Biopsychosocial model-based patient education

A BPS model-based PE was developed as a physical therapist-led program consisting of lectures and exercises. Because individual patient education may be more effective for pain than group education [18], lectures and exercises were conducted individually using PowerPoint. Patient education was conducted using metaphors to facilitate patient understanding [19].

The PE included content on TKA and psychological factors, neuroscience, sleep, nutrition, and physical activity (Table 1). The session on TKA and psychological factors provided an orientation to the PE and provided information on the pathophysiology of knee OA and the TKA procedure. The aim of the session was to help participants understand the need to address CPSP caused by psychological factors, such as negative emotions, anxiety, and pain catastrophizing, in addition to the nociceptive pain associated with surgery. The neuroscience session provided information on neurophysiology while utilizing the patient version of the neurophysiology of pain test [20]. We explained that nerves are alarm systems that transmit information from tissues to the brain; we explained that CPSP may be caused not only by tissue damage but also by increased nerve sensitivity. We also explained the need for adherence to an analgesic medication to prevent hyperalgesia (including central sensitization syndrome [CSS]). Sleep sessions included an understanding of non-REM and REM sleep and the potential impact of poor sleep quality on CPSP. In addition, mindfulness was implemented as a relaxation technique, specifically incorporating techniques such as focused breathing, body scan meditation, and mindful awareness of sensations. The nutrition session explained that malnutrition may be associated with inflammation, glial cell activation, and oxidative stress, whereas the Mediterranean diet, which is rich in vegetables, beans, fruit, nuts, cereals, fish, and olive oil may reduce inflammation. In addition, we explained that dietary management and weight loss could help prevent CPSP. During the physical activity session, we emphasized that inactivity is associated with pain and that increasing the frequency of activity may reduce pain. We also explained that paying attention to your 24-hour movement behavior (reducing the time of sedentary behavior, increasing the time of physical activity, and getting enough sleep) prevents pain. Finally, we explained the possibility of eliminating inactivity by engaging in social interactions and connecting with others.

**Table 1:** Biopsychosocial model-based patient education. CPSP, chronic post-surgical pain; OA, osteoarthritis; REM, rapid eye movement; TKA, total knee arthroplasty.

Session	Lecture topics	Comprehension test
Pain and psychological factors	(1) Pathophysiology of knee OA, TKA, and progress of pain after TKA; (2) fear avoidance model; (3) cognitive reconstruction	(1) Characteristics of people prone to pain; (2) how to cope with psychological factors
Pain and neuroscience	(1) Pain-reducing substances; (2) sensitization; (3) medication	(1) Pain-reducing substances; (2) appropriate timing of medication
Pain and sleep	(1) REM and non-REM sleep; (2) sleep quality; (3) breathing	(1) Ideal sleep time; (2) how to improve sleep quality; (3) causes of poor sleep quality
Pain and nutrition	(1) Glial cells; (2) inflammation; (3) oxidative stress; (4) obesity; (5) Mediterranean diet	(1) Diet to reduce pain and inflammation; (2) diet that leads to CPSP
Pain and physical activity	(1) Intensity of physical activity; (2) physical activity and disease; (3) sedentary behavior; (4) relationships with people	(1) Benefits of physical activity; (2) pacing and self-management

The PE consisted of a total of five sessions; the first session was conducted on the third postoperative day, and the second, third, fourth, and fifth sessions were conducted individually within 11 days of TKA. Each session was no longer than 60 min. The physical therapist responsible for the PE was trained by a physical therapist specializing in pain. Patients were questioned about their understanding after each lecture and were re-educated if they did not fully understand the content of the session.

The control and PE groups both underwent conventional physical therapy, including range of motion exercises, muscle strength training, gait training using the orthosis and cane if necessary, and cold therapy. Physical therapy was started on Day 1 after TKA. The PE group attended all five sessions.

Variables

The primary outcome measure was the change in pain three months after TKA. Pain was evaluated using the change in Knee injury and Osteoarthritis Outcome Score (KOOS) pain score from before TKA to three months after TKA.

CSS, sleep disturbances, pain catastrophizing, anxiety, depression, marital status, and educational background; characteristics associated with pain were examined as well [21-25]. CSS was assessed using the Central Sensitization Inventory (CSI) [26]. The CSI consists of two parts: Part A (0-100 points) and Part B. Part A consists of 25 questions related to health conditions related to CSS, and Part B asks whether the patient has a history of diseases characteristic of CSS. In this study, only Part A was used, and the higher the score, the more severe the CSS. The CSI shows a high degree of internal consistency and excellent agreement in test-retest reliability [27]. We used the Pittsburgh Sleep Quality Index (PSQI) to assess sleep disturbances [28]. The PSQI consists of a total of 18 questions about sleep over the past month, consisting of open questions and Likert scales. It has good internal consistency [29], and the overall PSQI score ranges from 0-21, with higher scores indicating more disturbed sleep.

Pain catastrophizing was measured using the Pain Catastrophizing Scale (PCS) [30]. This scale comprises 13 items, each of which was measured using a five-test method. The overall PCS score ranged from 0-52, with higher scores indicating more severe pain catastrophizing. We used the Japanese version of the PCS, which is highly reliable and has been previously validated [31]. The Hospital Anxiety and Depression Scale (HADS) consists of seven items related to anxiety (HADS-Anxiety; HADS-A) and seven items related to depression (HADS-Depression; HADS-D), which are answered on a 4-point scale of 0-3 [32]. The HADS-A and HADS-D are each scored between 0 and 21, and the higher the score, the more likely it is that the patient has anxiety and depression. Marital status was evaluated as being married or not at the time of preoperative TKA. Educational background was classified as the completion of compulsory education (elementary school and junior high school in Japan) or completion of further education after completing compulsory education.

Sample size

The required sample size was calculated using G*Power software (v. 3.1.9.6 Heinrich-Heine-Universität Düsseldorf, Düsseldorf, Germany). Assuming a comparison between two paired groups after propensity score matching with an alpha-error of 0.05, a statistical power of 0.8, and an effect size of 0.5 (moderate), at least 34 patients were needed in each group. The alpha was set to <0.05 to avoid causing type I errors [33]. The power was set at 0.80 because the risk of a type II error is greater when the power is <0.80 [33]. The effect size was arbitrarily set at 0.5 (moderate) because the effect size of similar interventions has not been clarified in previous studies.

Statistical analysis

Patients were categorized into the control and PE groups. Baseline normality for age, height, weight, body mass index (BMI), KOOS pain, CSI, PSQI, PCS, HADS-A, and HADS-D scores was confirmed before propensity score matching using the Shapiro-Wilk test. Unpaired t-tests were used to compare groups of data with normal distribution and Mann-Whitney U tests were performed for data with non-normal distributions. Preoperative factors associated with pain (age, sex, BMI, KOOS pain, CSI, PSQI, PCS, HADS-A, HADS-D scores, marital status, and education background) were transformed into propensity scores as covariates. Then, one-to-one propensity score matching was performed to reduce or minimize selection and confounding bias. Many confounding factors were matched and analyzed simultaneously using one-to-one propensity score matching, to exclude unmatched cases and create similar groups as in a randomized experiment [34]. Propensity score matching was performed using nearest neighbor matching with a caliper of 0.2 without replacement. The matching quality was evaluated using the c-statistic. If the c-statistic was between 0.6 and 0.9, propensity score analysis was applied [35]. The c-statistic for the propensity score model was 0.70, indicating good discrimination between patients in the control and PE groups. After propensity score matching, baseline normality of age, height, weight, BMI, CSI, PSQI, PCS, HADS-A, and HADS-D scores in the control and PE groups was confirmed using the Shapiro-Wilk test. Paired t-tests were used to compare groups of data with normal distribution and Wilcoxon signed-rank tests were performed for data with non-normal distribution. The chi-square test was used to compare sex, Kellgren-Lawrence grade, marital status, and educational background at baseline before and after propensity score matching between the control and PE groups. All baseline items were measured one day before TKA. The changes in KOOS pain, CSI, PCS, PSQI, HADS-A, and HADS-D scores from the preoperative period to three months postoperatively in the control and PE groups were compared after propensity score matching. The significance level was set at 5%. All statistical data were analyzed using SPSS ver. 29.0 statistical software (IBM Corp., Armonk, NY).

## Results

The demographic variables and preoperative BPS factors of the participants before and after propensity score matching are presented in Table 2. Propensity score matching was performed on 71 subjects in the control group and 50 subjects in the PE group, resulting in 37 subjects in each group for the statistical analyses. No significant differences in demographic and preoperative BPS factors were observed between the groups. Propensity score matching minimized differences in most variables.

**Table 2:** Comparison of patient characteristics at baseline between control and PE groups. Data are shown as means (standard derivation) or n (%). Abbrevations: n, number; BMI, body mass index; CSI, Central Sensitization Inventory; HADS-A, Hospital Anxiety and Depression Scale-Anxiety; HADS-D, Hospital Anxiety and Depression Scale-Depression; K-L grade, Kellgren-Lawrence grade; KOOS, Knee injury and Osteoarthritis Outcome Score; PCS, Pain Catastrophizing Scale; PE, patient education; PSQI, Pittsburgh Sleep Quality Index. ^a^ unpaired t-test;^ b^ Mann-Whitney U test; ^c^ chi-square test; ^d^ paired t-test; ^e^ Wilcoxon signed-rank test.

Variables	Pre-matched					Post-matched			
	Control group (n = 71)	PE group (n = 50)	*P*-value	Effect size		Control group (n = 37)	PE group (n = 37)	*P*-value	Effect size
Age (years)	75 (7.3)	74 (7.5)	0.443^b^	*r* = 0.070		74 (7.8)	75 (7.5)	0.745^d^	*r* = 0.050
Sex, female, n (%)	57 (80)	40 (80)	0.969^c^	*φ* = 0.003		28 (76)	30 (81)	0.572^c^	*φ* = 0.066
Height (cm)	152.4 (8.0)	153.9 (8.4)	0.354^a^	*r* = 0.080		152.4 (7.9)	153.3 (8.7)	0.611^d^	*r* = 0.090
Weight (kg)	60.8 (10.9)	60.9 (11.8)	0.955^a^	*r *= 0.010		60.1 (10.7)	61.4 (12.2)	0.629^d^	*r* = 0.080
BMI (kg/m^2^)	26.1 (3.9)	25.6 (3.5)	0.434^a^	*r *= 0.070		25.8 (3.5)	26.0 (3.7)	0.804^d^	*r* = 0.040
KOOS pain	49.1 (13.0)	50.9 (14.1)	0.483^a^	*r *= 0.060		49.6 (13.1)	49.6 (13.2)	0.979^d^	*r* = 0.000
CSI	20.7 (11.4)	19.2 (11.2)	0.402^b^	*r *= 0.076		18.5 (10.3)	21.0 (12.0)	0.396^e^	*r *= 0.077
PSQI	6.7 (2.5)	6.9 (3.2)	0.804^b^	*r *= 0.023		6.8 (2.5)	7.0 (3.4)	0.799^e^	*r* = 0.023
PCS	21.4 (9.6)	24.7 (9.6)	0.067^a^	*r *= 0.170		21.3 (9.7)	23.3 (10.2)	0.401^e^	*r* = 0.076
HADS-A	4.9 (2.1)	4.6 (2.9)	0.517^b^	*r *= 0.059		4.6 (2.3)	5.0 (3.1)	0.599^e^	*r* = 0.480
HADS-D	4.7 (2.6)	5.2 (3.2)	0.535^b^	*r *= 0.056		4.9 (2.6)	5.0 (3.0)	0.867^e^	*r* = 0.015
K-L grade, n (%)	Grade I: 0; grade II: 0; grade III: 17 (24); grade IV: 54 (76)	Grade I: 0; grade II: 0; grade III: 13 (26); grade IV: 37 (74)	0.796^c^	*φ *= 0.023		Grade I: 0; grade II: 0; grade III: 11 (30); grade IV: 26 (70)	Grade I: 0; grade II: 0; grade III: 10 (27); grade IV: 27 (73)	0.797^c^	*φ* = 0.030
Marital status; person with a spouse, n (%)	54 (76)	36 (72)	0.615^c^	*φ *= 0.046		29 (78)	27 (73)	0.588^c^	*φ* = 0.063
Education background, n (%)	Compulsory education: 10 (14); post-compulsory education: 61 (86)	Compulsory education: 9 (18); post-compulsory education: 41 (82)	0.560^c^	*φ *= 0.053		Compulsory education: 6 (16); post-compulsory education: 31 (84)	Compulsory education: 7 (19); post-compulsory education: 30 (81)	0.760^c^	*φ* = 0.036

Table 3 shows the changes in each parameter from before TKA to three months after TKA in the control and PE groups after propensity score matching. CSI (*p* = 0.041, effect size: *r* = 0.238), PSQI (*p* = 0.040, *r* = 0.239), and PCS (*p* = 0.004, *r* = 0.334) scores improved significantly in the PE group compared with the control group. There was no significant difference in KOOS pain (*p* = 0.143,* r* = 0.240) scores between the two groups, although a small effect size was obtained. No significant differences in HADS-A (*p* = 0.233,* r* = 0.139) and HADS-D (*p* = 0.333,* r* = 0.113) scores were detected between the two groups. 

**Table 3:** Comparison of changes in outcomes between the control and PE groups. Data are shown as means (standard derivation). Abbreviations: CSI, Central Sensitization Inventory; HADS-A, Hospital Anxiety and Depression Scale-Anxiety; HADS-D, Hospital Anxiety and Depression Scale-Depression; KOOS, Knee injury and Osteoarthritis Outcome Score; PCS, Pain Catastrophizing Scale; PE, patient education; PSQI, Pittsburgh Sleep Quality Index. ^a^ paired t-test; ^b^ Wilcoxon signed-rank test

Variables	Post-matched			
	Control group (n = 37)	PE group (n = 37)	*P*-value	Effect size
KOOS pain	20.1 (14.8)	24.2 (10.9)	0.143^a^	*r* = 0.240
CSI	− 2.4 (6.0)	− 5.3 (4.3)	0.041^b^	*r* = 0.238
PSQI	− 0.4 (1.4)	− 1.3 (2.1)	0.040^b^	*r *= 0.239
PCS	− 4.8 (9.1)	− 12.2 (9.2)	0.004^b^	*r* = 0.334
HADS-A	− 0.8 (2.0)	− 1.7 (2.8)	0.233^b^	*r *= 0.139
HADS-D	− 1.1 (2.1)	− 1.7 (2.7)	0.333^b^	*r *= 0.113

## Discussion

We developed a PE that included content focused on sleep and nutrition, based on the BPS model for post-TKA patients. We investigated the effectiveness of PE in reducing pain. PE had a small effect size in reducing pain after TKA. Early and individualized postoperative PE that includes content on sleep and nutrition, based on the BPS model, may help improve CSS, sleep disturbance, and pain catastrophizing after TKA.

The novelty of this study was that the inclusion of sleep and nutritional content in PE based on the BPS model showed the potential to improve CSS, sleep disturbance, and pain catastrophizing at three months after TKA. Patient education to date has included preoperative interventions for TKA but not interventions during the postoperative period when nociceptive pain is present [12]. Furthermore, while existing BPS model-based interventions improved pain catastrophizing [36], the effects of these interventions on CPSP after TKA were not clear. The results of this study will help clinicians determine treatment options for managing pain after TKA.

Although PE improved multiple pain-related factors such as CSS, sleep disturbance, and pain catastrophizing through a comprehensive approach to pain in the BPS model, the effect size for pain reduction was small. It may be that PE alone, which focuses on sleep and nutritional management and is effective for reducing nociceptive pain, does not improve pain at three months after TKA. Although patient education on nutrition has been provided, changes in nutritional status were not assessed. Therefore, nutrition-related assessments should be conducted to establish effective interventions for CPSP prevention. It has been shown that CSS, sleep disturbance, and pain catastrophizing can affect pain after three months after TKA [23, 37, 38]. Evaluating pain after three months or more after TKA may clarify the effects of PE in more detail.

The strength of this study was that it developed a new PE based on the BPS model, which includes sleep and nutrition. Furthermore, the study evaluated the effects of PE on multiple pain-related risk factors in addition to pain. Nevertheless, this study has certain limitations. First, the nutritional status was not assessed. Nutritional status should be assessed to determine if nutritional management can contribute to the reduction of nociceptive pain. Second, the PE group interacted with the physical therapist longer than patients in the control group. In the future, the effectiveness of the PE group in reducing pain should be compared with that of the control group receiving the same amount of intervention. Third, medication use was not assessed. Medication use may influence pain. Future studies should consider the type and duration of drug use. Fourth, the sample size may be insufficient to generalize the results. Although the sample size was sufficient to demonstrate a moderate effect size, the current study population may not represent the entire population of patients with pain after TKA. Therefore, a larger study is needed to increase the generalizability of this study.

## Conclusions

A PE program based on a BPS model was developed and its effects on pain were determined. The results demonstrate that the PE had a small effect on pain reduction, but significantly improved CSS, sleep disturbance, and pain catastrophizing. However, no significant effects on anxiety and depression were detected. These findings may help clinicians determine how to improve pain and pain-related risk factors after TKA.

## References

[1] Rice DA, Kluger MT, McNair PJ (2018). Persistent postoperative pain after total knee arthroplasty: a prospective cohort study of potential risk factors. Br J Anaesth.

[2] Beswick AD, Wylde V, Gooberman-Hill R, Blom A, Dieppe P (2012). What proportion of patients report long-term pain after total hip or knee replacement for osteoarthritis? A systematic review of prospective studies in unselected patients. BMJ Open.

[3] Schug SA, Lavand'homme P, Barke A, Korwisi B, Rief W, Treede RD (2019). The IASP classification of chronic pain for ICD-11: chronic postsurgical or posttraumatic pain. Pain.

[4] Aso K, Ikeuchi M, Takaya S (2021). Chronic postsurgical pain after total knee arthroplasty: A prospective cohort study in Japanese population. Mod Rheumatol.

[5] Jette DU, Hunter SJ, Burkett L (2020). Physical therapist management of total knee arthroplasty. Phys Ther.

[6] Scott CE, Howie CR, MacDonald D, Biant LC (2010). Predicting dissatisfaction following total knee replacement: a prospective study of 1217 patients. J Bone Joint Surg Br.

[7] Whale K, Wylde V, Beswick A, Rathbone J, Vedhara K, Gooberman-Hill R (2019). Effectiveness and reporting standards of psychological interventions for improving short-term and long-term pain outcomes after total knee replacement: a systematic review. BMJ Open.

[8] Booth J, Moseley GL, Schiltenwolf M, Cashin A, Davies M, Hübscher M (2017). Exercise for chronic musculoskeletal pain: A biopsychosocial approach. Musculoskeletal Care.

[9] Hajihasani A, Rouhani M, Salavati M, Hedayati R, Kahlaee AH (2019). The influence of cognitive behavioral therapy on pain, quality of life, and depression in patients receiving physical therapy for chronic low back pain: a systematic review. PM R.

[10] Victor L, Richeimer SM (2003). Psychosocial therapies for neck pain. Phys Med Rehabil Clin N Am.

[11] Wijma AJ, van Wilgen CP, Meeus M, Nijs J (2016). Clinical biopsychosocial physiotherapy assessment of patients with chronic pain: The first step in pain neuroscience education. Physiother Theory Pract.

[12] Louw A, Puentedura EJ, Reed J, Zimney K, Grimm D, Landers MR (2019). A controlled clinical trial of preoperative pain neuroscience education for patients about to undergo total knee arthroplasty. Clin Rehabil.

[13] Luo D, Fan Z, Yin W (2024). Chronic post-surgical pain after total knee arthroplasty: a narrative review. Perioper Med (Lond).

[14] Chouchou F, Khoury S, Chauny JM, Denis R, Lavigne GJ (2014). Postoperative sleep disruptions: a potential catalyst of acute pain?. Sleep Med Rev.

[15] Varallo G, Giusti EM, Manna C, Castelnuovo G, Pizza F, Franceschini C, Plazzi G (2022). Sleep disturbances and sleep disorders as risk factors for chronic postsurgical pain: A systematic review and meta-analysis. Sleep Med Rev.

[16] Nishimoto J, Shiraoka T, Takiguchi Y (2023). Derivation of a clinical prediction rule for chronic post-surgical pain after total knee arthroplasty considering biopsychosocial factors: A prospective cohort study. Knee.

[17] Bjørklund G, Aaseth J, Doşa MD, Pivina L, Dadar M, Pen JJ, Chirumbolo S (2019). Does diet play a role in reducing nociception related to inflammation and chronic pain?. Nutrition.

[18] Moseley GL (2003). Joining forces - combining cognition-targeted motor control training with group or individual pain physiology education: a successful treatment for chronic low back pain. J Man Manip Ther.

[19] Louw A, Puentedura EJ, Diener I, Zimney KJ, Cox T (2019). Pain neuroscience education: Which pain neuroscience education metaphor worked best?. S Afr J Physiother.

[20] Catley MJ, O'Connell NE, Moseley GL (2013). How good is the neurophysiology of pain questionnaire? A Rasch analysis of psychometric properties. J Pain.

[21] Kim SH, Yoon KB, Yoon DM, Yoo JH, Ahn KR (2015). Influence of centrally mediated symptoms on postoperative pain in osteoarthritis patients undergoing total knee arthroplasty: a prospective observational evaluation. Pain Pract.

[22] Chen AF, Orozco FR, Austin LS, Post ZD, Deirmengian CA, Ong AC (2016). Prospective evaluation of sleep disturbances after total knee arthroplasty. J Arthroplasty.

[23] Lewis GN, Rice DA, McNair PJ, Kluger M (2015). Predictors of persistent pain after total knee arthroplasty: a systematic review and meta-analysis. Br J Anaesth.

[24] Roubion RC, Fox RS, Townsend LA, Pollock GR, Leonardi C, Dasa V (2016). Does material status impact outcomes after total knee arthroplasty?. J Arthroplasty.

[25] Núñez-Cortés R, Chamorro C, Ortega-Palavecinos M, Mattar G, Paredes O, Besoaín-Saldaña Á, Cruz-Montecinos C (2019). Social determinants associated to chronic pain after total knee arthroplasty. Int Orthop.

[26] Mayer TG, Neblett R, Cohen H (2012). The development and psychometric validation of the central sensitization inventory. Pain Pract.

[27] Tanaka K, Nishigami T, Mibu A (2017). Validation of the Japanese version of the Central Sensitization Inventory in patients with musculoskeletal disorders. PLoS One.

[28] Buysse DJ, Reynolds CF 3rd, Monk TH, Berman SR, Kupfer DJ (1989). The Pittsburgh Sleep Quality Index: a new instrument for psychiatric practice and research. Psychiatry Res.

[29] Doi Y, Minowa M, Uchiyama M, Okawa M, Kim K, Shibui K, Kamei Y (2000). Psychometric assessment of subjective sleep quality using the Japanese version of the Pittsburgh Sleep Quality Index (PSQI-J) in psychiatric disordered and control subjects. Psychiatry Res.

[30] Sullivan MJ, Bishop SR, Pivik J (1995). The pain catastrophizing scale: development and validation. Psychol Assess.

[31] Matsuoka H, Sakano Y (2007). Assessment of cognitive aspect of pain: development, reliability, and validation of Japanese version of pain catastrophizing scale. Jpn J Psychosom Med.

[32] Zigmond AS, Snaith RP (1983). The hospital anxiety and depression scale. Acta Psychiatr Scand.

[33] Cohen J (1992). A power primer. Psychol Bull.

[34] D'Agostino RB Jr (1998). Propensity score methods for bias reduction in the comparison of a treatment to a non-randomized control group. Stat Med.

[35] Stürmer T, Joshi M, Glynn RJ, Avorn J, Rothman KJ, Schneeweiss S (2006). A review of the application of propensity score methods yielded increasing use, advantages in specific settings, but not substantially different estimates compared with conventional multivariable methods. J Clin Epidemiol.

[36] Bhatia S, Karvannan H, Prem V (2020). The effect of bio psychosocial model of rehabilitation on pain and quality of life after total knee replacement: a randomized controlled trial. J Arthrosc Joint Surg.

[37] Kim MS, Kim JJ, Kang KH, Lee JH, In Y (2024). Central sensitization and neuropathic pain cumulatively affect patients reporting inferior outcomes following total knee arthroplasty. J Bone Joint Surg Am.

[38] Edwards RR, Campbell C, Schreiber KL (2022). Multimodal prediction of pain and functional outcomes 6 months following total knee replacement: a prospective cohort study. BMC Musculoskelet Disord.

